# Longitudinal Assessment of Blood-Based Inflammatory, Neuromuscular, and Neurovascular Biomarker Profiles in Intensive Care Unit–Acquired Weakness: A Prospective Single-Center Cohort Study

**DOI:** 10.1007/s12028-024-02050-x

**Published:** 2024-07-09

**Authors:** Felix Klawitter, Friederike Laukien, Dagmar-C. Fischer, Anja Rahn, Katrin Porath, Lena Danckert, Rika Bajorat, Uwe Walter, Robert Patejdl, Johannes Ehler

**Affiliations:** 1 Department of Anesthesiology, Intensive Care Medicine, and Pain Therapy, Rostock University Medical Center, Rostock, 18057 Germany; 2 Department of Pediatrics, Rostock University Medical Center, Rostock, Germany; 3 Oscar Langendorff Institute of Physiology, Rostock University Medical Center, Rostock, Germany; 4 Department of Neurology, Rostock University Medical Center, Rostock, Germany; 5 Department of Medicine, Health and Medical University Erfurt, Erfurt, Germany; 6 Department of Anesthesiology and Intensive Care Medicine, University Hospital Jena, Jena, Germany

**Keywords:** Muscle weakness, Critical illness, Biomarkers, Neuromuscular diseases, Prospective study

## Abstract

**Background:**

The diagnosis of intensive care unit (ICU)-acquired weakness (ICUAW) and critical illness neuromyopathy (CINM) is frequently hampered in the clinical routine. We evaluated a novel panel of blood-based inflammatory, neuromuscular, and neurovascular biomarkers as an alternative diagnostic approach for ICUAW and CINM.

**Methods:**

Patients admitted to the ICU with a Sequential Organ Failure Assessment score of ≥ 8 on 3 consecutive days within the first 5 days as well as healthy controls were enrolled. The Medical Research Council Sum Score (MRCSS) was calculated, and motor and sensory electroneurography (ENG) for assessment of peripheral nerve function were performed at days 3 and 10. ICUAW was defined by an MRCSS < 48 and CINM by pathological ENG alterations, both at day 10. Blood samples were taken at days 3, 10, and 17 for quantitative analysis of 18 different biomarkers (white blood cell count, C-reactive protein, procalcitonin, C-terminal agrin filament, fatty-acid-binding protein 3, growth and differentiation factor 15, syndecan 1, troponin I, interferon-γ, tumor necrosis factor-α, interleukin-1α [IL-1α], IL-1β, IL-4, IL-6, IL-8, IL-10, IL-13, and monocyte chemoattractant protein 1). Results of the biomarker analysis were categorized according to the ICUAW and CINM status. Clinical outcome was assessed after 3 months.

**Results:**

Between October 2016 and December 2018, 38 critically ill patients, grouped into ICUAW (18 with and 20 without) and CINM (18 with and 17 without), as well as ten healthy volunteers were included. Biomarkers were significantly elevated in critically ill patients compared to healthy controls and correlated with disease severity and 3-month outcome parameters. However, none of the biomarkers enabled discrimination of patients with and without neuromuscular impairment, irrespective of applied classification.

**Conclusions:**

Blood-based biomarkers are generally elevated in ICU patients but do not identify patients with ICUAW or CINM.

*Trial registration***:** ClinicalTrials.gov identifier: NCT02706314.

**Supplementary Information:**

The online version contains supplementary material available at 10.1007/s12028-024-02050-x.

## Introduction

In critically ill patients, intensive care unit (ICU)-acquired weakness (ICUAW) is one of the most common causes of neuromuscular impairment. This is associated with increased morbidity, mortality, and long-term impairment in quality of life up to 5 years after ICU discharge [1]. In daily routine practice, clinical assessment and manual muscle strength testing using established scores such as the Medical Research Council Sum Score (MRCSS) represent the most frequently performed and recommended diagnostic tests for ICUAW [2–4]. However, prolonged sedation, mechanical ventilation, and unconsciousness due to neurological impairment frequently hampers clinical examination during the acute phase of critical illness [5]. Muscle biopsies and electroneurography (ENG) are established procedures for the diagnosis of critical illness neuromyopathy (CINM) but are rarely performed in daily clinical routine because of their invasiveness and required expertise [6]. Although ultrasonography can be considered as a valuable tool for the detection of neuromuscular pathologies, evidence with focus on ICUAW and CINM is still limited because of the absence of well-powered studies [7]. In contrast, the diagnostic and prognostic value of blood-based biomarkers has been widely evaluated in several disease entities within critically ill patients, including delirium [8, 9] and sepsis-associated encephalopathy [10]. Advantages of blood-based biomarkers include their easy assessment through simple blood sampling and the opportunity of serial measurements to monitor disease progression. In the field of ICUAW and CINM, the specific value of blood-based biomarkers for the detection and monitoring of ICUAW as well as the prediction of patient outcomes remains unclear. Because sepsis is considered one of the main risk factors for ICUAW, multiple cytokines, including tumor necrosis factor-α (TNFα), interleukin-1 (IL-1), IL-6, IL-10, and interferon-γ (IFNγ), have been suggested to promote muscle proteolysis, activate the ubiquitin proteasome system, induce contractile dysfunction, and inhibit muscle protein synthesis, but in contrast, they have also been suggested to enhance muscle regeneration following injury [11–16]. Cleavage products of vascular endothelium glycocalyx shredding in sepsis might therefore also be detectable in ICUAW [17, 18]. In case of CINM, direct markers of neuromuscular damage might include proteins released from the nerval and muscular compartments as well as from the interlinking neuromuscular junction following inflammation, immobilization, and mechanical silencing [19].

To overcome this gap, we conducted a prospective observational study using a broad panel of cytokines and inflammatory (white blood cell count, C-reactive protein, procalcitonin, IFNγ, TNFα, IL-1α, IL-1β, IL-4, IL-6, IL-8, IL-10, IL-13, monocyte chemoattractant protein 1, and growth and differentiation factor 15 [GDF15]), neurovascular (syndecan 1) and neuromuscular (C-terminal agrin filament [CAF], troponin I, and fatty-acid-binding protein 3 [FABP3]) biomarkers. We hypothesized, that distinct longitudinal biomarker profiles help to differentiate between patients with and without ICUAW and correlate with clinical outcome parameters.

## Methods

### Study design and inclusion and exclusion criteria

We conducted a prospective single-center observational cohort study. The study was performed in accordance with the Declaration of Helsinki and approved by the local ethics committee of the University of Rostock (ethics identifier: AS 2016–0016). Written informed consent for participation was given by healthy volunteers, patients, or a legal representative prior to enrollment. The present investigation represents the laboratory analysis part of a comprehensive and combined clinical–experimental study. Registration was made prior to the trial beginning and before the first patient enrollment (registered at ClinicalTrials.gov: NCT02706314). Results of the clinical and ex vivo experimental parts have already been published [20, 21]. Briefly, patients being at least 17 years of age and presenting with a Sequential Organ Failure Assessment (SOFA) score ≥ 8 on 3 consecutive days within the first 5 days after ICU admission were defined as critically ill and were eligible for enrollment. Main exclusion criteria comprised preexisting neuromuscular disorders and high-dosage corticosteroid treatment with ≥ 300 mg of hydrocortisone or equivalent per day. Further details are published elsewhere [18]. Clinical and neurophysiologic examinations were performed on days 3 and 10 after enrollment, and blood was sampled at study days 3, 10, and 17. The Barthel Index (BI) and the modified Rankin scale (mRS) 3 months after enrollment were considered as markers of the long-term patient outcome. Additionally, healthy volunteers without any preexisting neuromuscular disorders from the Institute of Physiology of the Rostock University Medical Center were recruited and received the same clinical and neurophysiological tests as the patients in one session. This trial was conceptualized as a pilot study.

### Clinical assessment and definition of ICUAW

Clinical evaluation of muscle strength was performed by the MRCSS assessment at study days 3 and 10. Intubated and ventilated patients received a sedation holiday prior to clinical examination. Patients were considered eligible for muscle strength testing using the MRCSS based on the score of five questions [22, 23]. ICUAW was considered by an MRCSS < 48 at day 10 after enrollment, and patients were subsequently defined as ICUAW positive (ICUAW +); otherwise, they were defined as ICUAW negative (ICUAW −) [24].

### Electrophysiology and definition of CINM

ENG was performed as described in detail before [21]. Briefly, at study days 3 and 10, compound motor action potentials were assessed over the musculus abductor digiti minimi and the musculus extensor digitorum brevis. Stimuli were applied via the stimulator module of an Epoch XP EMG machine (Axon Systems), and recordings of evoked responses were recorded at 10 kHz with 1-Hz high-pass and mains filtering using a PowerLab28T system (AD instruments, New Zealand) via surface EMG electrodes (Kendall, Covidien, Ireland). A nonpathological response was defined by a minimum amplitude of 4 mV. Sensory nerve action potentials were recorded using the antidromic technique over the superficial radial and the sural nerves with a threshold of 7.5 µV for a normal response in the averaged trace of ten repeated recordings. CINM was diagnosed as an absence of muscle reflexes, reduced muscle tone, and movements induced by painful stimuli as well as reduced compound motor action potentials and sensory nerve action potentials in four or more recording sites, and patients were defined as CINM positive (CINM +); otherwise, patients were defined as CINM negative (CINM −).

### Biomarker measurements in plasma and serum samples

Analyses were performed using commercially available enzyme-linked immunosorbent assay (ELISA) kits. The biomarker panel comprised skeletal muscle troponin I (catalog number: MBS765801, Human TNNI1 ELISA Kit, Mybiosource, San Diego, CA), FABP3 (catalog number: BMS 2263, Human FABP-3 ELISA, Thermo Fisher Scientific, Waltham, MA), and CAF (catalog number: MBS7606926, Human CAF ELISA Kit, Mybiosource, San Diego, CA). Syndecan 1 was used to assess vascular endothelium (catalog number: ab46506, Human Syndecan-1 ELISA Kit, Abcam, Cambridge, UK). Cytokine profiling comprised the stress-related GDF15 (catalog number: ab155432, GDF-15 Human ELISA Kit, abcam, Cambridge, UK) and inflammatory cytokines (catalog number: ab197449, Human inflammation Antibody Array A [IL-1α, IL1-β, IL-4, IL-6, IL-8, IL-10, IL-13, MCP1, IFNγ, TNFα] – Quantitative, abcam, Cambridge, UK). Plasma and serum samples were centrifuged at 4 °C with 2500 rpm for 15 min and aliquoted and stored at − 80 °C until analysis. All ELISA measurements were performed according to manufacturer-specific protocols, and all samples were assessed in duplicate.

### Statistics

Microsoft Excel 2010 (Microsoft, Redmond, WA) was used for data curation. Statistical analysis was done with IBM SPSS Statistics (Version 25, IBM Corp., Armonk, NY). The Shapiro–Wilk test was used for assessment of normal distribution of continuous data. Results are presented as frequency (percentage), median (interquartile range [IQR]), or mean (standard deviation) as appropriate. Student’s *t*-test, the Mann–Whitney *U*-test, Fisher’s exact test, and the χ^2^ test were employed. For correlation analysis between normally and nonnormally distributed data, the Pearson correlation coefficient and the Spearman rank correlation coefficient were computed, respectively. Statistical significance was indicated by *p* < 0.05. All statistical tests were two-sided.

## Results

### Study population characteristics and outcome data

**Fig. 1:**
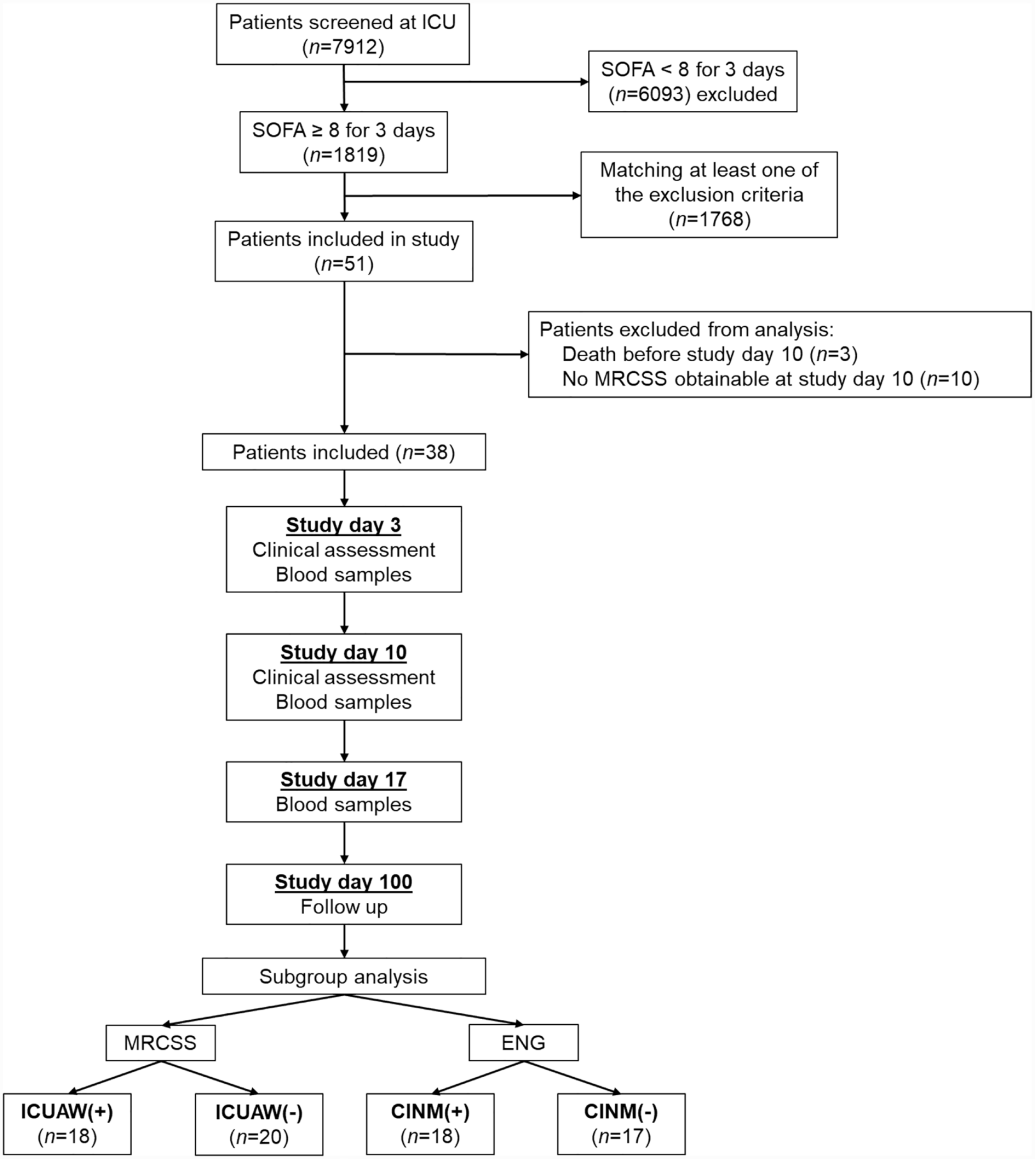
Study flowchart. CINM critical illness neuromyopathy, ENG electroneurography, ICU intensive care unit, ICUAW( +) patients with intensive-care-unit-acquired weakness, ICUAW( −) patients without intensive-care-unit-acquired weakness, MRCSS Medical Research Council Sum Score, SOFA Sequential Organ Failure Assessment score

**Fig. 2:**
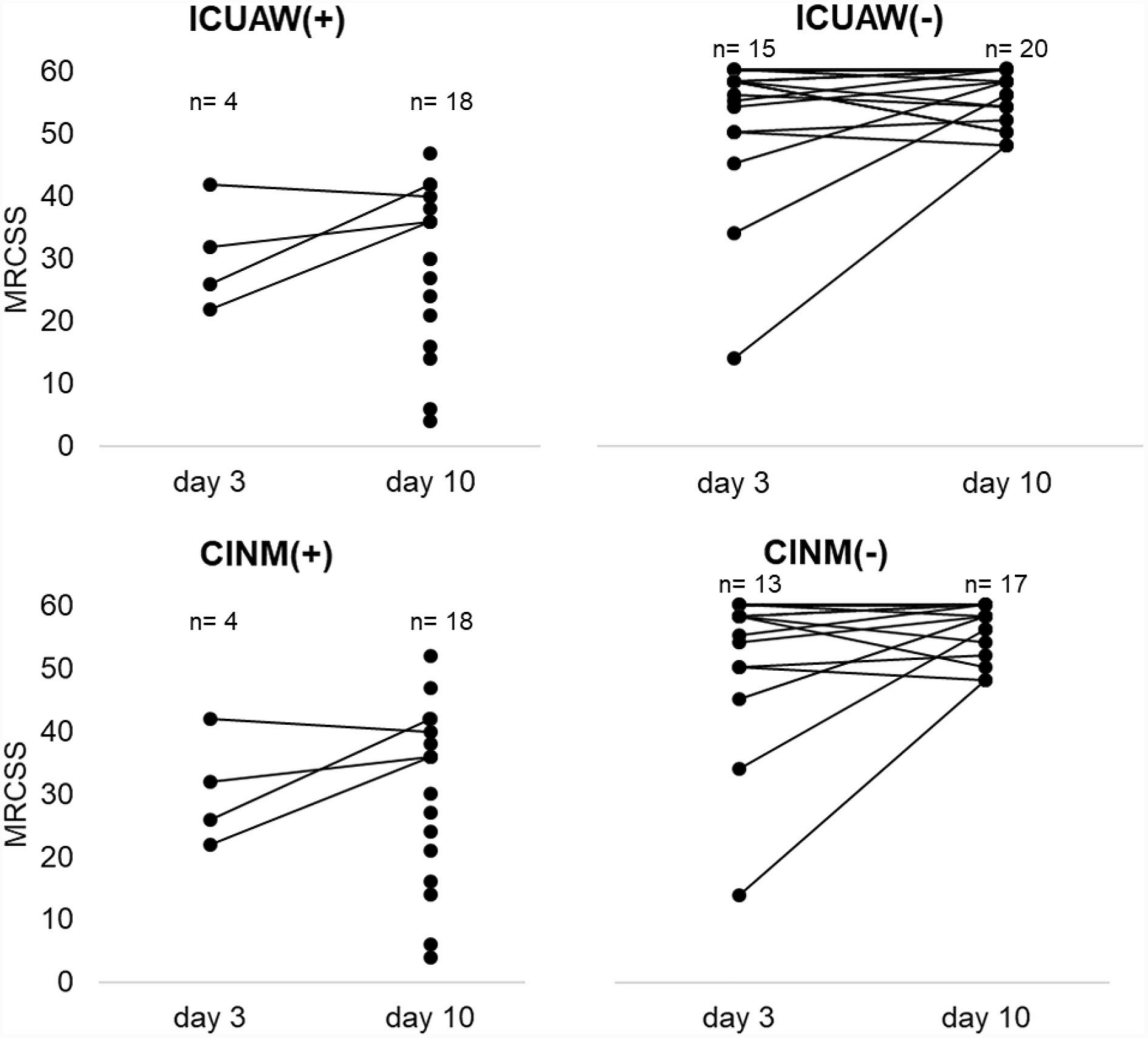
Muscle strength assessment. CINM critical illness neuromyopathy, ICUAW intensive-care-unit-acquired weakness, MRCSS Medical Research Council Sum Score

In total, 51 critically ill patients and ten healthy controls were recruited between October 2016 and December 2018 (Fig. 1). Three patients died within the first days after enrollment, and these data were therefore excluded from the analysis. Furthermore, the MRCSS at study day 10 was not assessable in ten patients because of prolonged sedation, delirium, or patient incompliance. Thus, data from 38 patients were available for subsequent analysis. Baseline characteristics of these patients are given in Table 1. Subsequently, patients were categorized according to the presence of ICUAW and CINM. All patients with ICUAW, except one with an MRCSS of 52 at study day 10, were also classified as CINM + . All patients, regardless of their classification, were comparable in terms of their activities of daily living and neuromuscular function prior to hospital admission. Because of inconclusive ENG data, we had to exclude three patients from the CINM subgroup analysis. We also collected data from ten healthy controls (six men, mean age 53.1 ± 8 years) without any preexisting neuromuscular disorder (Supplementary Table 1). Patients with and without ICUAW as well as with and without CINM were comparable for age, sex distribution, type of surgery, sepsis, and renal dysfunction. The SOFA scores and the MRCSS (Fig. 2) at study days 3 and 10 were significantly higher in patients with ICUAW and CINM compared with the corresponding negative groups. No differences in the degree of neurological impairment represented by the mRS and overall survival were observed between the groups. Only the BI 3 months after enrollment was significantly lower in the ICUAW + group (*p* = 0.03).

**Table 1:** Study population characteristics and outcome parameters

	ICUAW −	ICUAW +	*p* value	CINM −	CINM +	*p* value
Basic demographic data						
Total, *n* (%)	20 (42)	18 (38)	N/A	17 (49)	18 (51)	N/A
Male, *n* (%)	15 (75)	10 (56)	0.307	13 (77)	11 (61)	0.328
Age in years, mean (SD)	68.1 (14.2)	70.8 (11.5)	0.52	67.9 (15.2)	70.4 (11.6)	0.577
Cardiac and vascular surgery, *n* (%)	11 (55.0)	10 (55.6)	0.998	10 (59)	9 (50)	0.600
Thoracic surgery (noncardiac), *n* (%)	2 (10.0)	0 (0)	0.488	1 (6)	1 (6)	0.966
Visceral surgery, *n* (%)	3 (15.0)	5 (27.8)	0.438	2 (12)	4 (22)	0.411
Trauma surgery, *n* (%)	2 (10.0)	1 (5.5)	0.989	2 (12)	1 (6)	0.512
General surgery, *n* (%)	1 (5.0)	0 (0)	0.999	1 (6)	1 (6)	0.966
Urology, *n* (%)	1 (5.0)	0 (0)	0.999	1 (6)	0	0.485
Medical, *n* (%)	0 (0)	2 (11.0)	0.218	0	2 (11)	0.486
Sepsis, *n* (%)	3 (15)	4 (22)	0.687	3 (17)	0	0.104
APACHE II, mean (SD)	23.7 (6.2)	26.2 (3.7)	0.14	25.1 (4.9)	25.3 (5.7)	0.716
SOFA day 3, mean (SD)	10.1 (2.3)	12.9 (2.8)	**0.001**	10.1 (2.4)	12.6 (2.7)	**0.006**
SOFA day 10, mean (SD)	3.6 (3.0)	6.6 (2.8)	**0.001**	3.3 (2.5)	6.3 (2.9)	**0.001**
MRCSS day 3, mean (SD)	51.2 (12.4)	30.5 (8.7)	**0.01**	50.3 (13.1)	30.5 (8.7)	**0.017**
MRCSS day 10, mean (SD)	55.4 (4.2)	29.5 (12.9)	** < 0.0001**	56.0 (4.2)	30.7 (13.9)	** < 0.001**
Dialysis needed, *n* (%)	7 (35.0)	5 (27.8)	0.734	5 (29)	4 (22.2)	0.551
Creatinine (µmol/L) day 3, median (IQR)	126.0 (104.0–297.0)	114.5 (83.0–218.0)	0.362	125.5 (89.3–302.0)	114.5 (89.9–221.3)	0.617
Creatinine (µmol/L) day 10, median (IQR)	129.0 (74.1–275.0)	106.5 (76.7–182.3)	0.693	112.5 (64.3–297.0)	101.2 (70.8–180.8)	0.756
Creatinine (µmol/L) day 17, median (IQR)	129.5 (73.1–175.5)	86.5 (60.3–157.0)	0.315	131.0 (68.1–253.0)	86.4 (59.2–135.0)	0.169
Outcome parameters						
mRS after 3 months, mean (SD)	2.1 (2.6)	3.1 (2.2)	0.32	1.7 (2.5)	2.9 (2.2)	0.164
Barthel Index at admission, mean (SD)	97.5 (4.4)	93.9 (16.4)	0.68	98.2 (3.5)	93.4 (16.4)	1.0
Barthel Index after 3 months, mean (SD)	87.9 (25.5)	63.7 (36.0)	**0.03**	93.8 (13.2)	70.0 (32.2)	0.02
28-day survival, *n* (%)	18 (90)	18 (100)	0.49	15 (88)	18 (100)	0.134
3-month survival, *n* (%)	16 (80)	16 (88.9)	0.66	13 (77)	15 (83)	0.576

Significant *p* values are marked in bold

APACHE II, Acute Physiology and Chronic Health Evaluation II score, CINM, critical illness neuromyopathy, ICUAW, intensive-care-unit-acquired weakness, IQR, interquartile range, MRCSS, Medical Research Council Sum Score, mRS, modified Rankin Scale, N/A, not applicable, SOFA, Sequential Organ Failure Assessment score

### Skeletal muscle biomarker profiles

Blood levels of skeletal muscle biomarkers were similar between the subgroups at study days 3, 10, and 17 (Table 2). Only FABP3 levels tended to be higher in ICUAW − compared with ICUAW + patients but sharply missed significance (median 14,785 [IQR 5836–24,000] pg/mL vs. 4125 [IQR 1089–15,536] pg/mL, *p* = 0.059). Except for CAF, concentrations of FABP3, GDF15, syndecan 1, and skeletal muscle troponin I were significantly higher in ICUAW + and ICUAW − patients compared with healthy controls (Supplementary Table 2).

**Table 2:** Skeletal muscle biomarker levels

Muscle and endothelial biomarkers	ICUAW − , median (IQR)	ICUAW + , median (IQR)	*p* value	CINM − , median (IQR)	CINM + , median (IQR)	*p* value
CAF (pg/mL) day 3	106.5 (65.9–127.8)	152.5 (72.1–230.5)	0.120	97.8 (52.3–128.4)	129.5 (62.5–222.5)	0.143
CAF (pg/mL) day 10	79.1 (54.8–105.1)	72.4 (44.6–226.9)	0.937	78.9 (36.5–103.6)	72.4 (41.5–215.4)	0.853
CAF (pg/mL) day 17	76.1 (25.9–105.4)	90.5 (15.6–311.0)	0.452	76.1 (22.9–105.4)	70.2 (14.2–273.8)	0.730
FABP3 (pg/mL) day 3	34,525 (14,850–102,650)	25,900 (17,103–87,050)	0.843	67,100 (10,650– 117,500)	30,050 (15,077–10,0025)	0.925
FABP3 (pg/mL) day 10	7047 (5055–31,125)	13,125 (5763–22,288)	0.640	5805 (3225–34,750)	13,125 (4594–26,413)	0.766
FABP3 (pg/mL) day 17	14,785 (5836–24,000)	4125 (1089–15,537)	0.059	16,910 (5836–33,300)	4125 (1195–16,350)	0.081
GDF15 (pg/mL) day 3	14,281 (4569–23,738)	13,448 (6213–27,813)	0.654	12,210 (4050–17,250)	8633 (5114–25,738)	0.752
GDF15 (pg/mL) day 10	7665 (3914–20,550)	10,610 (5168–22,556)	0.579	6630 (3,153–23,175)	8355 (4221–19,269)	0.644
GDF15 (pg/mL) day 17	11,799 (8545–16,288)	9790 (2939–13,475)	0.346	11,799 (8545–16,288)	7900 (2505–14,600)	0.245
Syndecan 1 (pg/mL) day 3	278.2 (206.0–350.6)	310.0 (289.9–365.1)	0.384	294.7 (140.9–378.3)	306.2 (247.0–359.7)	0.857
Syndecan 1 (pg/mL) day 10	317.5 (224.3–379.2)	328.1 (279.7–378.8)	0.739	318.2 (206.9–379.4)	321.1 (243.1–377.8)	0.914
Syndecan 1 (pg/mL) day 17	369.5 (331.1–378.5)	374.1 (281.1–375.9)	0.764	369.5 (205.5–378.5)	374.1 (306.1–376.3)	0.905
Troponin I (pg/mL) day 3	4.1 (3.5–4.7)	3.9 (3.6–4.2)	0.532	4.1 (3.3–4.7)	3.9 (3.4–4.4)	0.517
Troponin I (pg/mL) day 10	4.1 (3.6–4.6)	4.0 (3.5–4.2)	0.498	4.0 (3.2–4.8)	4.0 (3.5–4.4)	0.509
Troponin I (pg/mL) day 17	4.1 (3.8–4.4)	3.7 (3.5–4.4)	0.502	4.0 (3.3–4.4)	3.7 (3.4–4.5)	0.872

CAF, C-terminal agrin filament, CINM, critical illness neuromyopathy, FABP, fatty acid binding protein, GDF, growth and differentiation factor, ICUAW, intensive-care-unit-acquired weakness, IQR, interquartile range

### Cytokine and inflammatory biomarker profiles

Biomarker levels of both ICUAW + and ICUAW − patients were elevated in similar patterns compared with healthy controls (Supplementary Table 3). Except for IL-1α at study day 17 (median 16.7 [IQR 5.9–27.3] pg/mL vs. 7.9 [IQR 2.7–12.7] pg/mL, *p* = 0.023) in the ICUAW subgroup comparisons and IL-10 at study day 17 (median 4.7 [IQR 2.2–5.2] pg/mL vs. 1.8 [IQR 1.4–3.3] pg/mL, *p* = 0.038) in patients with and without CINM, we found no statistically significant differences in biomarker levels between the subgroups (Table 3).

**Table 3:** Cytokine and inflammatory biomarker levels

Cytokines and inflammatory biomarkers	ICUAW − , median (IQR)	ICUAW + , median (IQR)	*p* value	CINM − , median (IQR)	CINM + , median (IQR)	*p* value
CRP (mg/L) day 3	202.0 (105.0–272.5)	213.0 (129.0–243.3)	0.788	201.0 (75.1–316.5)	216.5 (119.1–259.8)	0.558
CRP (mg/L) day 10	121.0 (75.2–193.0)	68.9 (45.1–118.5)	0.068	110.3 (74.–05.8)	73.5 (39.0–135.3)	0.120
WBC (10^9^/L) day 3	11.9 (9.1–18.2)	14.0 (9.1–16.2)	0.989	11.3 (8.6–18.1)	12.9 (8.8–16.4)	0.741
WBC (10^9^/L) day 10	12.3 (9.4–17.9)	13.4 (11.1–16.4)	0.726	12.1 (8.6–18.5)	13.4 (10.8–16.8)	0.448
PCT (ng/mL) day 3	1.0 (0.4–4.9)	2.5 (1.0–9.0)	0.193	0.9 (0.4–6.2)	2.5 (0.9–10.6)	0.181
PCT (ng/mL) day 10	0.2 (0.1–0.8)	0.3 (0.2–0.7)	0.334	0.2 (0.1–0.8)	0.3 (0.2–0.8)	0.235
IFNγ (pg/mL) day 3	1.9 (1.1–2.3)	1.6 (1.1–1.8)	0.579	1.8 (0.8–2.3)	1.5 (1.0–2.1)	0.564
IFNγ (pg/mL) day 10	1.7 (1.2–2.5)	1.5 (1.0–2.7)	0.812	1.7 (1.0–2.5)	1.7 (1.0–2.8)	0.801
IFNγ (pg/mL) day 17	1.7 (0.6–2.4)	1.2 (0.6–2.0)	0.460	1.4 (0.4–2.5)	1.5 (0.5–2.2)	0.616
IL-10 (pg/mL) day 3	3.3 (3.0–3.7)	2.6 (2.0–3.3)	0.099	3.4 (2.9–4.0)	2.6 (1.8–4.1)	0.122
IL-10 (pg/mL) day 10	2.9 (1.8–4.3)	2.2 (1.6–3.2)	0.366	2.9 (1.7–4.4)	2.2 (1.4–4.4)	0.517
IL-10 (pg/mL) day 17	4.0 (2.2–4.8)	1.7 (1.4–2.6)	0.079	4.7 (2.2–5.2)	1.8 (1.4–3.3)	**0.038**
IL-13 (pg/mL) day 3	2.1 (1.4–3.1)	1.5 (1.4–1.8)	0.188	2.0 (1.4–3.3)	1.6 (1.4–1.8)	0.387
IL-13 (pg/mL) day 10	1.9 (1.6–3.5)	1.6 (1.3–2.8)	0.358	1.8 (1.5–3.5)	1.8 (1.4–3.6)	0.759
IL-13 (pg/mL) day 17	2.4 (1.4–2.7)	1.5 (1.2–1.7)	0.089	2.4 (1.4–2.7)	1.5 (1.1–2.3)	0.192
IL-1α (pg/mL) day 3	5.4 (4.4–9.1)	5.7 (4.3–7.1)	0.558	5.4 (3.5–8.6)	5.8 (4.5–8.3)	0.857
IL-1α (pg/mL) day 10	6.7 (4.8–9.6)	5.5 (4.3–8.8)	0.334	6.4 (4.7–10.3)	5.5 (3.5–12.3)	0.494
IL-1α (pg/mL) day 17	16.7 (5.9–27.3)	7.9 (2.7–12.7)	**0.023**	16.8 (1.5–38.2)	8.1 (2.7–14.0)	0.307
IL-1β (pg/mL) day 3	2.9 (0.5–7.4)	1.4 (0.9–4.5)	0.716	2.3 (0.4–7.4)	1.5 (0.9–6.3)	0.792
IL-1β (pg/mL) day 10	2.0 (0.5–8.5)	2.1 (0.6–6.7)	0.899	1.7 (0.5–12.7)	3.0 (0.6–9.3)	0.603
IL-1β (pg/mL) day 17	7.9 (2.0–32.6)	5.0 (0.9–20.4)	0.571	7.9 (2.0–32.6)	9.9 (1.3–23.6)	0.941
IL-4 (pg/mL) day 3	2.0 (0.6–2.7)	1.6 (0.9–3.3)	0.974	1.2 (0.5–2.8)	1.7 (0.8–3.6)	0.624
IL-4 (pg/mL) day 10	2.5 (0.6–4.4)	1.0 (0.5–4.3)	0.486	2.2 (0.4–4.7)	1.3 (0.6–5.4)	0.943
IL-4 (pg/mL) day 17	2.3 (0.4–13.3)	1.5 (0.6–3.5)	0.378	2.3 (0.2–13.3)	2.4 (0.9–4.2)	0.941
IL-6 (pg/mL) day 3	12.0 (6.9–23.8)	10.4 (7.4–30.1)	0.643	14.2 (6.8–27.7)	10.4 (7.4–31.6)	0.880
IL-6 (pg/mL) day 10	9.3 (8.0–16.1)	8.4 (5.1–12.7)	0.231	9.3 (7.0–16.6)	8.5 (5.3–12.9)	0.553
IL-6 (pg/mL) day 17	13.6 (11.4–21.7)	9.4 (7.8–15.7)	0.144	13.6 (11.4–21.7)	11.6 (7.8–17.2)	0.217
IL-8 (pg/mL) day 3	9.4 (7.7–11.3)	10.2 (6.0–11.7)	0.526	10.0 (7.7–12.5)	7.7 (5.5–12.1)	0.214
IL-8 (pg/mL) day 10	10.1 (7.9–12.3)	8.3 (5.3–10.0)	0.178	10.1 (7.6–12.5)	8.6 (5.2–13.5)	0.296
IL-8 (pg/mL) day 17	16.6 (13.3–28.9)	11.3 (9.0–20.0)	0.245	16.6 (13.3–29.6)	11.3 (8.2–22.2)	0.192
MCP1 (pg/mL) day 3	138.7 (76.0–218.9)	82.8 (61.8–262.4)	0.579	156.4 (89.1–234.5)	77.3 (58.1–266.6)	0.280
MCP1 (pg/mL) day 10	109.0 (67.5–158.6)	60.8 (43.2–119.4)	0.261	138.0 (56.9–192.6)	67.5 (43.2–152.0)	0.249
MCP1 (pg/mL) day 17	217.9 (197.4–275.0)	229.3 (168.7–290.0)	0.913	217.9 (197.4–275.0)	229.3 (168.5–309.3)	0.916
TNFα (pg/mL) day 3	4.3 (3.7–4.8)	4.7 (3.1–5.6)	0.669	4.1 (3.4–4.9)	4.7 (3.1–5.7)	0.589
TNFα (pg/mL) day 10	4.4 (3.3–5.5)	4.5 (3.2–6.5)	0.837	4.2 (3.3–5.4)	5.0 (3.2–8.2)	0.428
TNFα (pg/mL) day 17	5.3 (4.1–12.3)	4.3 (1.6–7.2)	0.250	5.3 (4.1–12.6)	5.8 (1.2–7.6)	0.597

Significant *p* values are marked in bold

CINM, critical illness neuromyopathy, CRP, C-reactive protein, ICUAW, intensive-care-unit-acquired weakness, IQR, interquartile range, IFN, interferon, IL, interleukin, MCP1, monocyte chemoattractant protein 1, PCT, procalcitonin, TNF, tumor necrosis factor

### Longitudinal assessment of biomarker profiles

**Fig. 3:**
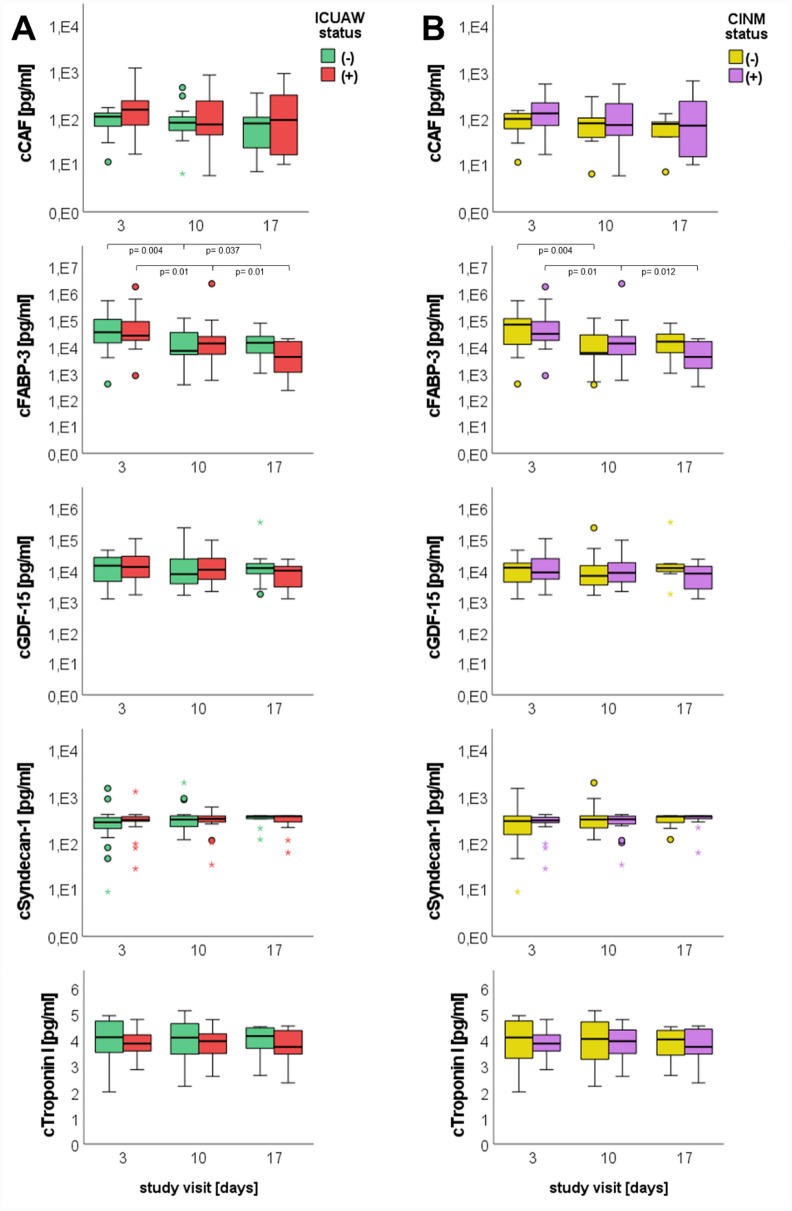
Longitudinal assessment of blood-based biomarker levels. **a**, Comparison between patients with (red box plots) and without (green box plots) ICUAW. **b**, Comparison between patients with (purple box plots) and without (yellow box plots) CINM. Box plots depict the median and the first and third quartiles, whiskers show values within the 1.5 × interquartile range. Dots represent values between 1.5 × and 3.0 × and stars values beyond the 3.0 × of the interquartile range. CAF C-terminal agrin filament, CINM critical illness neuromyopathy, FABP-3 fatty-acid-binding protein 3, GDF-15 growth and differentiation factor 15, ICUAW intensive-care-unit-acquired weakness (Color figure online)

Results from longitudinal monitoring of skeletal muscle biomarker profiles are presented in Fig. 3. FABP3 levels decreased significantly in both ICUAW + and CINM + subgroups until study day 17. In contrast, biomarker concentrations tended to increase between study days 10 and 17 in ICUAW − and CINM − patients. Similar patterns were observed for GDF15 but without statistical significance over time.

### Correlations of biomarker levels in patients with and without ICUAW

**Fig. 4:**
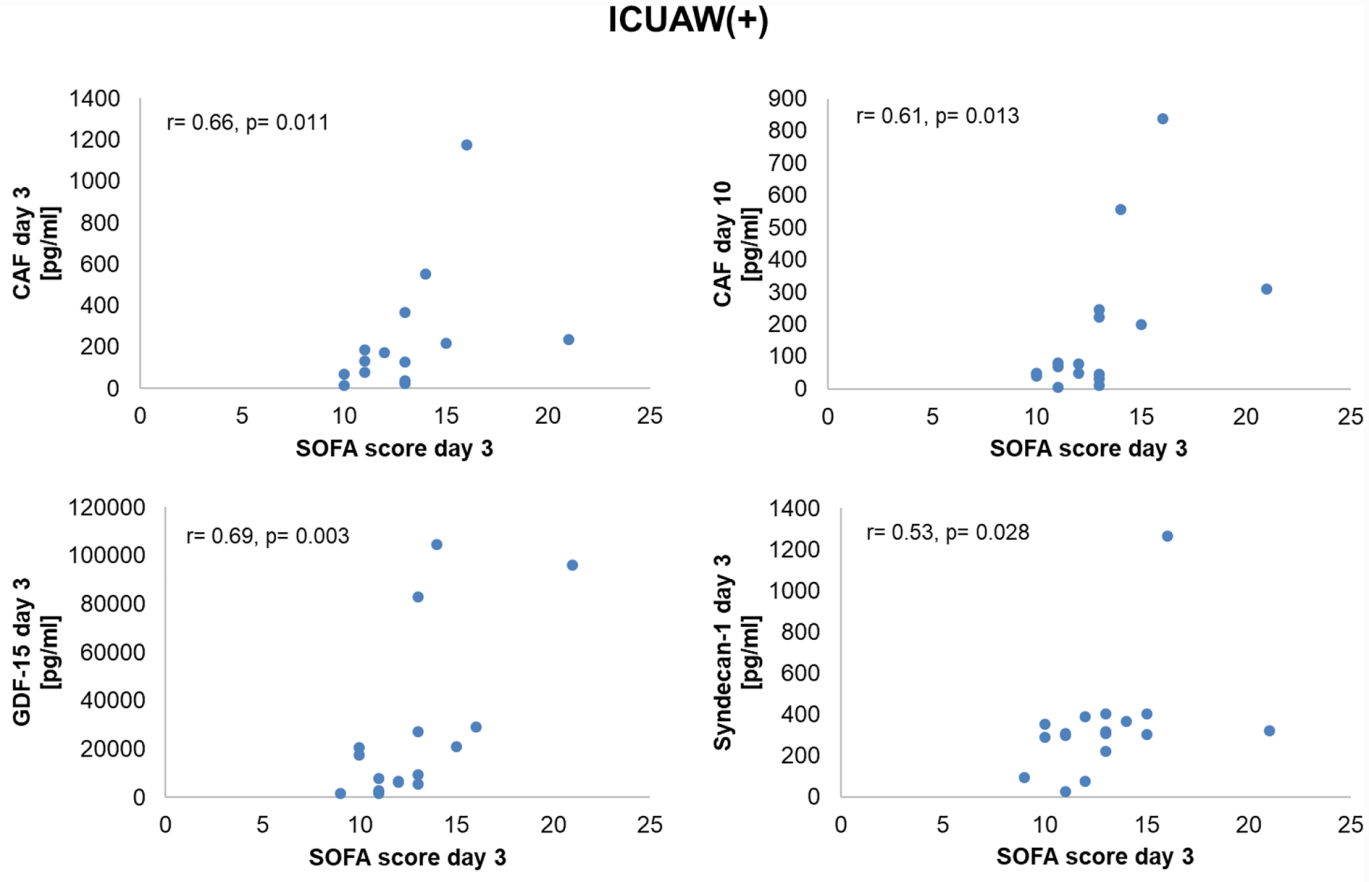
Correlation of biomarker levels with disease severity in ICUAW( +) patients. CAF C-terminal agrin filament, GDF-15 growth and differentiation factor 15, ICUAW intensive-care-unit-acquired weakness, SOFA Sequential Organ Failure Assessment

**Fig. 5:**
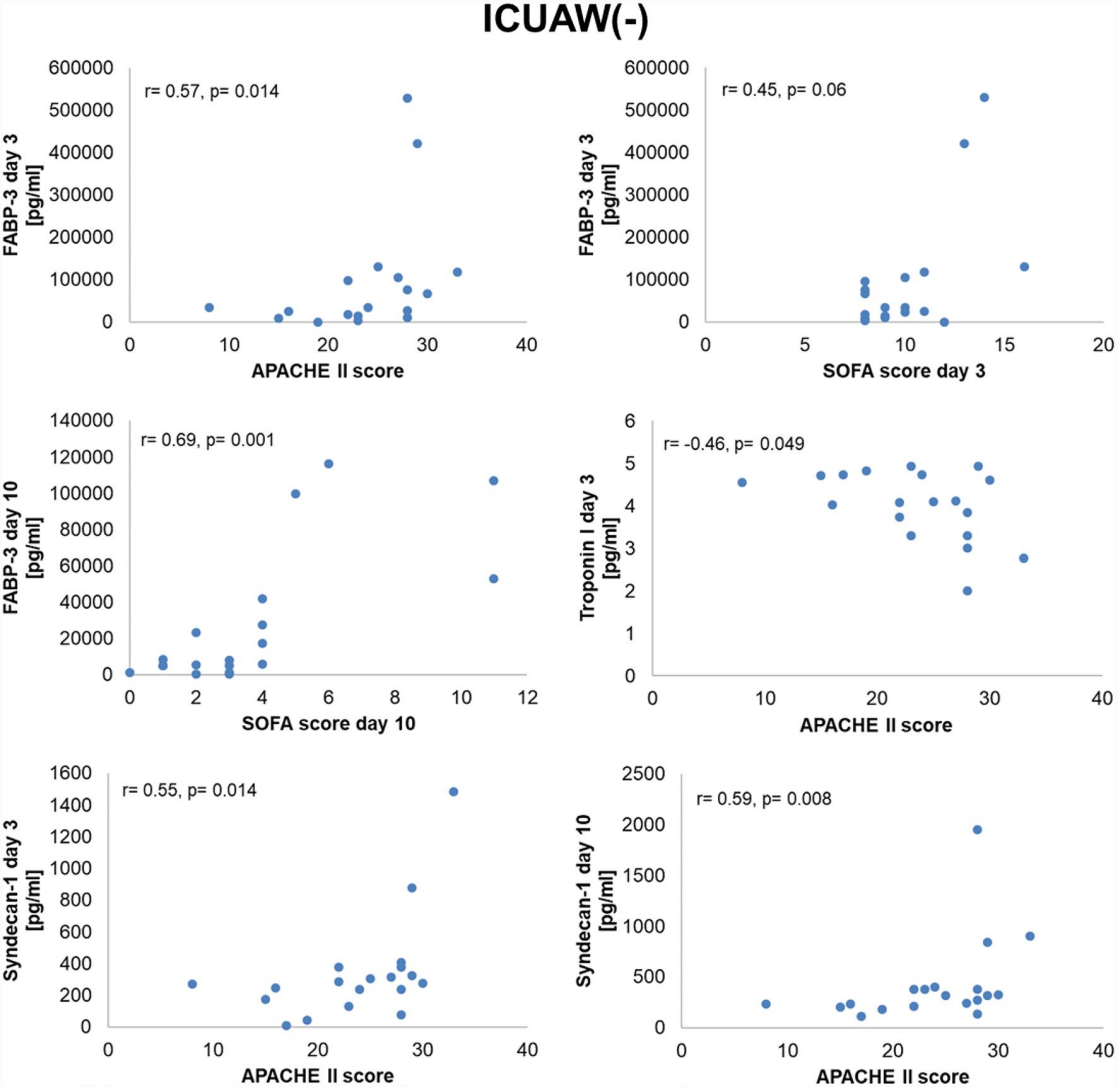
Correlation of biomarker levels with disease severity in ICUAW( −) patients. APACHE Acute Physiology and Chronic Health Evaluation, FABP-3 fatty-acid-binding protein 3, ICUAW intensive-care-unit-acquired weakness, SOFA Sequential Organ Failure Assessment

Skeletal muscle biomarkers correlated positively with measures of disease severity in both study groups (Figs. 4 and 5). In ICUAW + patients, GDF15, CAF, and syndecan 1 levels correlated with the early SOFA score at day 3. Similarly, multiple positive correlations with overall disease severity assessments and outcome parameters in ICUAW − patients were observed. FABP3 at day 3 correlated with the Acute Physiology and Chronic Health Evaluation (APACHE II) score and the SOFA score. Elevated syndecan 1 serum concentrations were associated with a higher APACHE II score. Conversely, skeletal muscle troponin I was negatively correlated with the APACHE II score. Neither in ICUAW + nor in ICUAW − patients were significant correlations found between skeletal muscle biomarkers and muscle strength.

A correlation analysis revealed multiple moderate to strong correlations of inflammatory biomarkers with distinct measures of disease severity. IL-4 levels at study day 3 as well as IL-1β levels at study day 10 correlated with the SOFA score at days 3 and 10 (IL-4: *r* = 0.52, *p* = 0.037; IL-1β: *r* =  − 0.64, *p* = 0.014) in ICUAW + patients. In contrast, in ICUAW − patients, IL-13 levels were negatively correlated with the APACHE II score at all study days (day 3: *r* =  − 0.40, *p* = 0.09; day 10: *r* =  − 0.60; *p* = 0.007; day 17: *r* =  − 0.71, *p* = 0.033).

### Correlations of biomarker levels in patients with and without CINM

Similar to the ICUAW subgroups, skeletal muscle biomarker levels of CINM + patients correlated with the SOFA score at day 3 (CAF day 3: *r* = 0.58, *p* = 0.03; FABP3 day 17: *r* = 0.57, *p* = 0.025; GDF15 day 3: *r* = 0.67, *p* = 0.005) and day 10 (CAF day 17: *r* = 0.59, *p* = 0.034) as well as with the BI at 3 months (FABP3 day 10: *r* =  − 0.53, *p* = 0.041). In CINM − patients, we observed multiple correlations of biomarker levels with outcome data, including the APACHE II score (FABP3 day 3: *r* = 0.58, *p* = 0.024; GDF15 day 10: *r* = 0.51, *p* = 0.036; syndecan 1 day 3: *r* = 0.59, *p* = 0.017; syndecan 1 day 10: *r* = 0.59, *p* = 0.016), the mRS at day 100 (GDF15 day 3: *r* = 0.69, *p* = 0.008; GDF15 day 10: *r* = 0.62, *p* = 0.015), and the SOFA score at day 10 (FABP3 day 10: *r* = 0.74, *p* < 0.001; FABP3 day 17: *r* = 0.80, *p* = 0.015; GDF15 day 3: *r* = 0.77, *p* = 0.003; GDF15 day 10: *r* = 0.86, *p* < 0.001; GDF15 day 17: *r* = 0.743, *p* = 0.035; syndecan 1 day 3: *r* = 0.55, *p* = 0.026; syndecan 1 day 10: *r* = 0.56, *p* = 0.025). Regarding the inflammatory biomarkers, no relevant correlations between biomarker levels and outcome parameters were observed.

## Discussion

We investigated the diagnostic value of a novel blood-based biomarker panel to differentiate between critically ill patients with and without acquired neuromuscular weakness. Skeletal muscle and inflammatory biomarkers are elevated in critically ill patients compared with healthy controls but do not discriminate between patients with and without ICUAW or CINM. Comparison of absolute biomarker concentrations between groups and longitudinal changes of blood biomarker levels over the course of 17 days revealed similar patterns in patients with and without ICUAW and CINM, despite clinically significant differences of overall limb muscle strength. Importantly, both subgroups were comparable for age, sex distribution, and renal dysfunction, which represent typical confounding factors in biomarker analysis. Nevertheless, other factors might have contributed to these results. First, although the MRCSS has been proven to be a valid and reliable clinical tool for the assessment of overall muscle strength in critically ill patients [25], a detailed differentiation between predominant neuropathy and/or myopathy is not possible and requires more elaborate methods [2]. Therefore, the reduction in muscle strength within the ICUAW + group might be due to a higher proportion of patients with predominant CIP rather than direct muscle damage. Therefore, we additionally performed ENG examinations, which showed similar observations. Unfortunately, serum biomarkers of neuroaxonal damage, such as neurofilament light and heavy chains, have not been assessed within the present trial but also showed no clear benefit in the detection of patients with ICUAW in former studies [26]. Second, except for troponin I, which is an integral part of the sarcomere troponin complex, no other structural components of the actual skeletal muscle contractile apparatus have been assessed as potential serum biomarkers here. Myosin filaments are known to be severely affected in ICUAW and CINM by insufficient protein synthesis and enhanced protein degradation, even within the first days after the onset of critical illness [27]. However, proteins within other structural and functional compartments of the skeletal muscle might be more resilient to these early alterations. FABP3, also known as heart-type fatty-acid-binding protein, is abundantly expressed in cardiomyocytes and slow-type skeletal muscle fibers [28]. Elevated serum levels of FAPB3 have been observed in polymyositis/dermatomyositis [29] and generalized sarcopenia [30]. Hereby, recent animal data point toward a critical role of FABP3 for muscle atrophy and endothelial dysfunction [31, 32]. These results might point toward a possible role of FABP3 in the development of sepsis-induced skeletal muscle microvascular dysfunction, potentially contributing to CIM [33]. In our study, serum levels of FABP3 were markedly elevated in both subgroups at the first assessment but progressively declined in ICUAW + and CINM + patients over time. In contrast, FABP3 serum levels elevated again significantly in ICUAW − and CINM − patients at study day 17. However, to date, evidence regarding the significance of FABP3 in critical-illness-induced muscle weakness and muscle atrophy is lacking.

CAF is an integral component of the neuromuscular junction and has been targeted as a potential serum biomarker in age-related sarcopenia, muscle disuse, and chronic muscle wasting and after stroke [34, 35]. Within the present study, serum CAF levels tended to be higher in patients with ICUAW compared to the ICUAW − group early during critical illness but without statistical significance, indicating similar neuromuscular impairment.

In a study by Bloch et al. [36], serum levels of GDF15 protein and GDF15 muscle mRNA were elevated in 20 critically ill patients with ICUAW compared with healthy controls, and the GDF15 serum levels of about 7000 pg/mL are comparable to the results in the present study. In contrast, Xie et al. [37] compared serum GDF15 in 50 patients with ICUAW and 45 patients without ICUAW and found significantly higher levels of serum GDF15 in patients with clinically relevant neuromuscular weakness after 7 days of critical illness. In our study, GDF15 levels were not different within both subgroups, but serum levels were up to sixfold higher compared to the results by Xie and coworkers [37]. This might be explained by the overall high disease severity of our study cohort, with markedly elevated APACHE II and SOFA scores compared to former studies.

Cytokines and biomarkers of inflammation also revealed no differences between patients with and without ICUAW or CINM. A possible explanation might be the similar proportion of patients with sepsis in both groups, indicating a comparable degree of systemic inflammation, regardless of underlying neuromuscular dysfunction. As for the skeletal muscle biomarker panel, we found no relevant correlations between serum cytokine levels and muscle strength, but we did find relevant correlations with disease severity scores. These results are comparable with a study by Winkelman et al. [38], who investigated IL-8, IL-15, and TNFα serum levels in critically ill patients and observed a positive association between IL-8 concentrations after mobilization and physical activity scores after ICU discharge but not with muscle strength.

A major strength of the present study is the investigation of a broad panel of inflammatory, neurovascular, and neuromuscular biomarkers. In total, we measured 18 different blood-based biomarkers, which is, to our knowledge, the largest biomarker panel ever assessed in the context of ICUAW and CINM. Furthermore, biomarker levels were longitudinally measured over 17 days after ICU admission, which allowed us to compare acute and subacute changes of biomarker concentrations related to the clinical outcome. Another advantage of the present study is the combined clinical and electrophysiological assessment of patients, providing two subgroup comparisons of biomarker data. However, some limitations have to be mentioned. Because of the relatively small study cohort, the present results may not have reached sufficient statistical power to show possible significant differences in biomarker levels between the individual groups. However, a power analysis was not performed, as the study was conceptualized as a pilot trial. Although multiple group comparisons at different study visits were calculated, we did not apply a statistical correction of the *p* value for multiple testing (e.g., a Bonferroni correction) because all comparisons were already statistically insignificant. Furthermore, despite good validation in ICU patients, the MRCSS remains a subjective measure, assessing skeletal muscle strength only in part and depending on the operator’s experience as well as patients’ compliance. Other tests such as dynamometry might have provided a more detailed assessment of overall muscle strength, but this assessment would also be dependent on patients’ cooperation, which supports the main idea of the present study to identify blood-based biomarkers independently indicating neuromuscular impairment. Furthermore, baseline assessment of patients before the onset of critical illness was not possible because of the study design.

## Conclusions

Blood-based inflammatory, neurovascular, and neuromuscular biomarkers are frequently elevated in critically ill patients but cannot distinguish between patients with and without acquired neuromuscular weakness. As far as we can conclude from our analysis, biomarker levels are not helpful to identify and monitor patients with ICUAW, although a certain correlation with overall disease severity was observed. Other diagnostic tools such as neuromuscular ultrasound might offer better opportunities in the future and should be analyzed in large-scale studies [7].

## Supplementary Information

Below is the link to the electronic supplementary material.

Supplementary file1 (DOCX 15 KB)

Supplementary file2 (DOCX 15 KB)

Supplementary file3 (DOCX 17 KB)

## Data Availability

Data are available on reasonable request.

## Clinical Trial Registration

ClinicalTrials.gov identifier: NCT02706314.

## References

[1] Vanhorebeek I, Latronico N, Van den Berghe G. ICU-acquired weakness. Intensive Care Med. 2020;46(4):637–53. 32076765 10.1007/s00134-020-05944-4 PMC7224132

[2] Latronico N, Rasulo FA, Eikermann M, Piva S. Illness weakness, polyneuropathy and myopathy: diagnosis, treatment, and long-term outcomes. Crit Care. 2023;27(1):439. Erratum in: Crit Care. 2023;27(1):469. 10.1186/s13054-023-04676-3 PMC10644573 37957759

[3] Klawitter F, Schaller SJ, Söhle M, Reuter DA, Ehler J. Intensive care unit-acquired weakness: a nationwide survey on diagnostics, monitoring and treatment strategies on German intensive care units. Anaesthesiologie. 2022;71(8):618–25. 35112164 10.1007/s00101-022-01089-9 PMC9352631

[4] Turan Z, Topaloglu M, Ozyemisci TO. Medical Research Council-sumscore: a tool for evaluating muscle weakness in patients with post-intensive care syndrome. Crit Care. 2020;24(1):562. 32948221 10.1186/s13054-020-03282-x PMC7499929

[5] Wu Y, Zhang Z, Jiang B, Wang G, Wei H, Li B, et al. Current practice and barriers to ICU-acquired weakness assessment: a cross-sectional survey. Physiotherapy. 2021;112:135–42. 34052568 10.1016/j.physio.2021.01.002

[6] Klawitter F, Oppitz MC, Goettel N, Berger MM, Hodgson C, Weber-Carstens S, et al. A global survey on diagnostic, therapeutic and preventive strategies in intensive care unit-acquired weakness. Medicina (Kaunas). 2022;58(8):1068. 36013535 10.3390/medicina58081068 PMC9416039

[7] Klawitter F, Walter U, Axer H, Ehler J. Intensive care unit-acquired weakness-diagnostic value of neuromuscular ultrasound. Anaesthesiologie. 2023;72(8):543–54. 37310449 10.1007/s00101-023-01300-5

[8] Ruhnau J, Müller J, Nowak S, Strack S, Sperlich D, Pohl A, et al. Serum biomarkers of a pro-neuroinflammatory state may define the pre-operative risk for postoperative delirium in spine surgery. Int J Mol Sci. 2023;24(12):10335. 37373482 10.3390/ijms241210335 PMC10298968

[9] Saller T, Petzold A, Zetterberg H, Kuhle J, Chappell D, von Dossow V, et al. A case series on the value of tau and neurofilament protein levels to predict and detect delirium in cardiac surgery patients. Biomed Pap Med Fac Univ Palacky Olomouc Czech Repub. 2019;163(3):241–6. 31530945 10.5507/bp.2019.043

[10] Bircak-Kuchtova B, Chung HY, Wickel J, Ehler J, Geis C. Neurofilament light chains to assess sepsis-associated encephalopathy: are we on the track toward clinical implementation? Crit Care. 2023;27(1):214. 37259091 10.1186/s13054-023-04497-4 PMC10230136

[11] De Larichaudy J, Zufferli A, Serra F, Isidori AM, Naro F, Dessalle K, et al. TNF-α- and tumor-induced skeletal muscle atrophy involves sphingolipid metabolism. Skelet Muscle. 2012;2(1):2. 22257771 10.1186/2044-5040-2-2 PMC3344678

[12] Reid MB, Lännergren J, Westerblad H. Respiratory and limb muscle weakness induced by tumor necrosis factor-alpha: involvement of muscle myofilaments. Am J Respir Crit Care Med. 2002;166(4):479–84. 12186824 10.1164/rccm.2202005

[13] Wondergem R, Graves BM, Li C, Williams DL. Lipopolysaccharide prolongs action potential duration in HL-1 mouse cardiomyocytes. Am J Physiol Cell Physiol. 2012;303(8):C825–33. 22895260 10.1152/ajpcell.00173.2012 PMC3469715

[14] Cooney RN, Maish GO 3rd, Gilpin T, Shumate ML, Lang CH, Vary TC. Mechanism of IL-1 induced inhibition of protein synthesis in skeletal muscle. Shock. 1999;11(4):235–41. 10220298 10.1097/00024382-199904000-00002

[15] Llovera M, Carbó N, López-Soriano J, García-Martínez C, Busquets S, Alvarez B, et al. Different cytokines modulate ubiquitin gene expression in rat skeletal muscle. Cancer Lett. 1998;133(1):83–7. 9929164 10.1016/s0304-3835(98)00216-x

[16] Cheng M, Nguyen MH, Fantuzzi G, Koh TJ. Endogenous interferon-gamma is required for efficient skeletal muscle regeneration. Am J Physiol Cell Physiol. 2008;294(5):C1183–91. 18353892 10.1152/ajpcell.00568.2007

[17] Piotti A, Novelli D, Meessen JM, Ferlicca D, Coppolecchia S, Marino A, et al.; ALBIOS Investigators. Endothelial damage in septic shock patients as evidenced by circulating syndecan-1, sphingosine-1-phosphate and soluble VE-cadherin: a substudy of ALBIOS. Crit Care. 2021;25(1):113. 10.1186/s13054-021-03545-1 PMC7980645 33741039

[18] Patejdl R, Walter U, Rosener S, Sauer M, Reuter DA, Ehler J. Muscular ultrasound, syndecan-1 and procalcitonin serum levels to assess intensive care unit-acquired weakness. Can J Neurol Sci. 2019;46(2):234–42. 30739614 10.1017/cjn.2018.390

[19] Hermans G, Van den Berghe G. Clinical review: intensive care unit acquired weakness. Crit Care. 2015;19(1):274. 26242743 10.1186/s13054-015-0993-7 PMC4526175

[20] Klawitter F, Walter U, Patejdl R, Endler J, Reuter DA, Ehler J. Sonographic evaluation of muscle echogenicity for the detection of intensive care unit-acquired weakness: a pilot single-center prospective cohort study. Diagnostics (Basel). 2022;12(6):1378. 35741188 10.3390/diagnostics12061378 PMC9221760

[21] Patejdl R, Klawitter F, Walter U, Zanaty K, Schwandner F, Sellmann T, et al. A novel ex vivo model for critical illness neuromyopathy using freshly resected human colon smooth muscle. Sci Rep. 2021;11(1):24249. 34930954 10.1038/s41598-021-03711-z PMC8688412

[22] De Jonghe B, Bastuji-Garin S, Durand MC, Malissin I, Rodrigues P, Cerf C, et al.; Groupe de Réflexion et d’Etude des Neuromyopathies en Réanimation. Respiratory weakness is associated with limb weakness and delayed weaning in critical illness. Crit Care Med. 2007;35(9):2007–15. 10.1097/01.ccm.0000281450.01881.d8 17855814

[23] Kleyweg RP, van der Meché FG, Schmitz PI. Interobserver agreement in the assessment of muscle strength and functional abilities in Guillain-Barré syndrome. Muscle Nerve. 1991;14(11):1103–9. 1745285 10.1002/mus.880141111

[24] Stevens RD, Marshall SA, Cornblath DR, Hoke A, Needham DM, de Jonghe B, et al. A framework for diagnosing and classifying intensive care unit-acquired weakness. Crit Care Med. 2009;37(10 Suppl):S299-308. 20046114 10.1097/CCM.0b013e3181b6ef67

[25] Vanpee G, Hermans G, Segers J, Gosselink R. Assessment of limb muscle strength in critically ill patients: a systematic review. Crit Care Med. 2014;42(3):701–11. 24201180 10.1097/CCM.0000000000000030

[26] Wieske L, Witteveen E, Petzold A, Verhamme C, Schultz MJ, van Schaik IN, et al. Neurofilaments as a plasma biomarker for ICU-acquired weakness: an observational pilot study. Crit Care. 2014;18(1):R18. 24443841 10.1186/cc13699 PMC4057240

[27] Kanova M, Kohout P. Molecular mechanisms underlying intensive care unit-acquired weakness and sarcopenia. Int J Mol Sci. 2022;23(15):8396. 35955530 10.3390/ijms23158396 PMC9368893

[28] Li B, Syed MH, Khan H, Singh KK, Qadura M. The role of fatty acid binding protein 3 in cardiovascular diseases. Biomedicines. 2022;10(9):2283. 36140383 10.3390/biomedicines10092283 PMC9496114

[29] Zhang L, Zhou H, Peng Q, Jiang W, Qiao W, Wang G. Fatty acid binding protein 3 is associated with skeletal muscle strength in polymyositis and dermatomyositis. Int J Rheum Dis. 2017;20(2):252–60. 26891180 10.1111/1756-185X.12838

[30] Qaisar R, Karim A, Muhammad T, Shah I, Khan J. Prediction of sarcopenia using a battery of circulating biomarkers. Sci Rep. 2021;11(1):8632. 33883602 10.1038/s41598-021-87974-6 PMC8060253

[31] Lee SM, Lee SH, Jung Y, Lee Y, Yoon JH, Choi JY, et al. FABP3-mediated membrane lipid saturation alters fluidity and induces ER stress in skeletal muscle with aging. Nat Commun. 2020;11(1):5661. 33168829 10.1038/s41467-020-19501-6 PMC7653047

[32] Nguyen HC, Bu S, Nikfarjam S, Rasheed B, Michels DC, Singh A, et al. Loss of fatty acid binding protein 3 ameliorates lipopolysaccharide-induced inflammation and endothelial dysfunction. J Biol Chem. 2023;299(3): 102921. 36681124 10.1016/j.jbc.2023.102921 PMC9988587

[33] Kowalewska PM, Kowalewski JE, Milkovich SL, Sové RJ, Wang L, Whitehead SN, et al. Spectroscopy detects skeletal muscle microvascular dysfunction during onset of sepsis in a rat fecal peritonitis model. Sci Rep. 2022;12(1):6339. 35428849 10.1038/s41598-022-10208-w PMC9012880

[34] Monti E, Sarto F, Sartori R, Zanchettin G, Löfler S, Kern H, et al. C-terminal agrin fragment as a biomarker of muscle wasting and weakness: a narrative review. J Cachexia Sarcopenia Muscle. 2023;14(2):730–44. 36772862 10.1002/jcsm.13189 PMC10067498

[35] Scherbakov N, Knops M, Ebner N, Valentova M, Sandek A, Grittner U, et al. Evaluation of C-terminal agrin fragment as a marker of muscle wasting in patients after acute stroke during early rehabilitation. J Cachexia Sarcopenia Muscle. 2016;7(1):60–7. 27066319 10.1002/jcsm.12068 PMC4799857

[36] Bloch SA, Lee JY, Syburra T, Rosendahl U, Griffiths MJ, Kemp PR, et al. Increased expression of GDF-15 may mediate ICU-acquired weakness by down-regulating muscle microRNAs. Thorax. 2015;70(3):219–28. 25516419 10.1136/thoraxjnl-2014-206225 PMC4345798

[37] Xie Y, Liu S, Zheng H, Cao L, Liu K, Li X. Utility of plasma GDF-15 for diagnosis and prognosis assessment of ICU-acquired weakness in mechanically ventilated patients: prospective observational study. BioMed Res Int. 2020;2020:3630568. 32104689 10.1155/2020/3630568 PMC7036092

[38] Winkelman C, Johnson KD, Gordon N. Associations between muscle-related cytokines and selected patient outcomes in the ICU. Biol Res Nurs. 2015;17(2):125–34. 24875632 10.1177/1099800414532709 PMC4247813

