# Supplementary material for: Longitudinal Assessment of Blood-Based Inflammatory, Neuromuscular, and Neurovascular Biomarker Profiles in Intensive Care Unit–Acquired Weakness: A Prospective Single-Center Cohort Study
Source: Neurocrit Care. 2024 Jul 9;42(1):118–30. doi: 10.1007/s12028-024-02050-x (PMC11811256; doi:10.1007/s12028-024-02050-x)
Supplement: Supplementary file 1 — Supplementary file1 (DOCX 15 KB) [file 12028_2024_2050_MOESM1_ESM.docx]

**Supplementary Table 1:** Study population characteristics and outcome parameters of healthy controls compared to ICUAW subgroups. APACHE II: Acute Physiology and Chronic Health Evaluation II Score. CINM: Critical Illness Neuromyopathy. ICUAW: Intensive Care Unit-Acquired Weakness. MRCSS: Medical Research Council Sum Score. mRS: modified Rankin Scale. SOFA: Sequential Organ Failure Assessment Score.

|  | **Controls** | **p Value**  **ICUAW(-) vs. Controls** | **p Value**  **ICUAW(+) vs. Controls** |
| --- | --- | --- | --- |
| **Basic demographic data** |  |  |  |
| Total, n (%) | 10 (26) | N/A | N/A |
| Male, n (%) | 6 (60) | 0.431 | 0.820 |
| Age in years, mean (SD) | 53.1 (8.0) | **0.002** | **<0.0001** |
| Cardiac and vascular surgery, n (%) | N/A | N/A | N/A |
| Thoracic surgery (noncardiac), n (%) | N/A | N/A | N/A |
| Visceral surgery, n (%) | N/A | N/A | N/A |
| Trauma surgery, n (%) | N/A | N/A | N/A |
| General surgery, n (%) | N/A | N/A | N/A |
| Urology, n (%) | N/A | N/A | N/A |
| Medical, n (%) | N/A | N/A | N/A |
| Sepsis, n (%) | N/A | N/A | N/A |
| APACHE II, mean (SD) | N/A | N/A | N/A |
| SOFA day 3, mean (SD) | N/A | N/A | N/A |
| SOFA day 10, mean (SD) | N/A | N/A | N/A |
| MRCSS day 3, mean (SD) | 60 (0) | **<0.0001** | **<0.0001** |
| MRCSS day 10, mean (SD) | 60 (0) | **<0.0001** | **<0.0001** |
| Dialysis needed, n (%) | 0 (0) | 0.064 | 0.128 |
| Creatinine [µmol/l] day 3, median (IQR) | N/A | N/A | N/A |
| Creatinine [µmol/l] day 10, median (IQR) | N/A | N/A | N/A |
| Creatinine [µmol/l] day 17, median (IQR) | N/A | N/A | N/A |
| **Outcome parameters** |  |  |  |
| mRS after three months, mean (SD) | 0 (0) | **0.005** | **<0.0001** |
| Barthel Index at admission, mean (SD) | N/A | N/A | N/A |
| Barthel Index after three months, mean (SD) | N/A | N/A | N/A |
| 28-day survival, n (%) | 10 (100) | 0.54 | 1 |
| Three months survival, n (%) | 10 (100) | 0.27 | 0.52 |
