# Supplementary material for: Longitudinal Assessment of Blood-Based Inflammatory, Neuromuscular, and Neurovascular Biomarker Profiles in Intensive Care Unit–Acquired Weakness: A Prospective Single-Center Cohort Study
Source: Neurocrit Care. 2024 Jul 9;42(1):118–30. doi: 10.1007/s12028-024-02050-x (PMC11811256; doi:10.1007/s12028-024-02050-x)
Supplement: Supplementary file 2 — Supplementary file2 (DOCX 15 KB) [file 12028_2024_2050_MOESM2_ESM.docx]

**Supplementary Table 2:** Skeletal muscle biomarker levels of healthy controls compared to ICUAW subgroups. CAF: C-terminal agrin filament. CINM: Critical Illness Neuromyopathy. FABP: Fatty acid binding protein. GDF: Growth and differentiation factor. ICUAW: Intensive Care Unit-Acquired Weakness.

| **Muscle and endothelial biomarkers**  median (IQR) | **Controls** | **p value**  **ICUAW(-) vs Controls** | **p value**  **ICUAW(-) vs Controls**  **(age corrected)** | **p value**  **ICUAW(+) vs Controls** | **p value**  **ICUAW(+) vs Controls**  **(age corrected)** |
| --- | --- | --- | --- | --- | --- |
| CAF [pg/ml] day 3 | 62.7 (61.9;319.0) | 0.143 | 0.302 | 0.579 | 0.276 |
| CAF [pg/ml] day 10 | = | 0.965 | 0.549 | 0.804 | 0.458 |
| CAF [pg/ml] day 17 | = | 0.685 | 0.654 | 0.882 | 0.385 |
| FABP-3 [pg/ml] day 3 | 727.3 (403.6;1586.3) | **<0.001** | 0.210 | **<0.001** | 0.709 |
| FABP-3 [pg/ml] day 10 | = | **0.001** | 0.144 | **<0.001** | 0.399 |
| FABP-3 [pg/ml] day 17 | = | **0.001** | 0.134 | **0.027** | **0.022** |
| GDF-15 [pg/ml] day 3 | 568.0 (496.6;706.5) | **0.001** | **0.024** | **<0.001** | **0.005** |
| GDF-15 [pg/ml] day 10 | = | **<0.001** | 0.546 | **<0.001** | **0.002** |
| GDF-15 [pg/ml] day 17 | = | **<0.001** | 0.649 | **<0.001** | **0.023** |
| Syndecan-1 [pg/ml] day 3 | 8.6 (6.7;33.0) | **<0.001** | 0.101 | **<0.001** | **0.006** |
| Syndecan-1 [pg/ml] day 10 | = | **<0.001** | 0.104 | **<0.001** | **<0.001** |
| Syndecan-1 [pg/ml] day 17 | = | **<0.001** | **<0.001** | **<0.001** | **<0.001** |
| Troponin I [pg/ml] day 3 | 3.1 (2.5;3.5) | **0.004** | **0.002** | **0.005** | **0.034** |
| Troponin I [pg/ml] day 10 | = | **0.002** | **<0.001** | **0.005** | **0.019** |
| Troponin I [pg/ml] day 17 | = | **0.013** | **0.029** | **0.019** | 0.170 |
