# Supplementary material for: Longitudinal Assessment of Blood-Based Inflammatory, Neuromuscular, and Neurovascular Biomarker Profiles in Intensive Care Unit–Acquired Weakness: A Prospective Single-Center Cohort Study
Source: Neurocrit Care. 2024 Jul 9;42(1):118–30. doi: 10.1007/s12028-024-02050-x (PMC11811256; doi:10.1007/s12028-024-02050-x)
Supplement: Supplementary file 3 — Supplementary file3 (DOCX 17 KB) [file 12028_2024_2050_MOESM3_ESM.docx]

**Supplementary Table 3:** Cytokine and inflammatory biomarker levels in healthy controls compared to ICUAW subgroups. CINM: Critical Illness Neuromyopathy. CRP: C-reactive protein. ICUAW: Intensive Care Unit-Acquired Weakness. IFN: Interferon. IL: Interleukin. MCP: Monocyte Chemoattractant Protein-1. PCT: Procalcitonin. TNF: Tumor necrosis factor.

| **Cytokines and**  **inflammatory**  **Biomarkers**  median (IQR) | **Controls** | **p value**  **ICUAW(-) vs Controls** | **p value**  **ICUAW(-) vs Controls**  **(age corrected)** | **p value**  **ICUAW(+) vs Controls** | **p value**  **ICUAW(+) vs Controls**  **(age corrected)** |
| --- | --- | --- | --- | --- | --- |
| CRP [mg/l] day 3 | 3.0 (3.0;3.0) | **0.002** | **0.005** | **0.001** | **0.004** |
| CRP [mg/l] day 10 | = | NA | NA | NA | NA |
| WBC [10E9/l] day 3 | N/A | NA | NA | NA | NA |
| WBC [10E9/l] day 10 | N/A | NA | NA | NA | NA |
| PCT [ng/ml] day 3 | 0.1 (0.1;0.1) | **0.001** | 0.756 | 0.119 | 0.491 |
| PCT [ng/ml] day 10 | = | NA | NA | NA | NA |
| IFNγ [pg/ml] day 3 | 1.6 (0.5;2.4) | 0.509 | 0.416 | 0.763 | 0.397 |
| IFNγ [pg/ml] day 10 | = | 0.521 | 0.217 | 0.482 | 0.465 |
| IFNγ [pg/ml] day 17 | = | 0.746 | 0.803 | 0.874 | 0.645 |
| IL10 [pg/ml] day 3 | 1.2 (0.9;1.7) | **<0.001** | **<0.001** | **0.001** | 0.225 |
| IL10 [pg/ml] day 10 | = | **0.001** | **0.004** | **0.006** | 0.113 |
| IL10 [pg/ml] day 17 | = | **<0.001** | **0.036** | **0.025** | **0.007** |
| IL13 [pg/ml] day 3 | 1.9 (1.1;2.6) | 0.337 | 0.267 | 0.979 | 0.398 |
| IL13 [pg/ml] day 10 | = | 0.279 | **0.030** | 0.535 | 0.146 |
| IL13 [pg/ml] day 17 | = | 0.278 | 0.280 | 0.492 | 0.649 |
| IL1α [pg/ml] day 3 | 8.7 (4.6;12.8) | 0.383 | 0.156 | 0.192 | 0.378 |
| IL1α [pg/ml] day 10 | = | 0.582 | 0.444 | 0.292 | 0.543 |
| IL1α [pg/ml] day 17 | = | 0.462 | 0.192 | 0.471 | 0.646 |
| IL1β [pg/ml] day 3 | 9.6 (2.4;26.6) | 0.130 | 0.232 | **0.023** | 0.154 |
| IL1β [pg/ml] day 10 | = | 0.066 | 0.435 | 0.079 | 0.279 |
| IL1β [pg/ml] day 17 | = | 0.744 | 0.419 | 0.380 | 0.744 |
| IL4 [pg/ml] day 3 | 2.6 (2.1;3.5) | 0.100 | 0.708 | 0.111 | 0.358 |
| IL4 [pg/ml] day 10 | = | 0.595 | 0.948 | 0.162 | 0.997 |
| IL4 [pg/ml] day 17 | = | 0.847 | 0.075 | 0.306 | 0.714 |
| IL6 [pg/ml] day 3 | 6.6 (3.7;7.2) | **0.018** | **0.034** | **0.004** | 0.251 |
| IL6 [pg/ml] day 10 | = | **0.010** | 0.393 | 0.183 | 0.208 |
| IL6 [pg/ml] day 17 | = | **0.003** | 0.417 | **0.003** | 0.181 |
| IL8 [pg/ml] day 3 | 8.2 (6.9;8.4) | 0.169 | 0.686 | 0.616 | 0.129 |
| IL8 [pg/ml] day 10 | = | 0.103 | 0.725 | 0.688 | 0.054 |
| IL8 [pg/ml] day 17 | = | **0.002** | 0.061 | **0.020** | **0.002** |
| MCP1 [pg/ml] day 3 | 123.2 (103.6;138.9) | 0.646 | 0.529 | 0.581 | 0.619 |
| MCP1 [pg/ml] day 10 | = | 0.783 | 0.794 | 0.071 | 0.393 |
| MCP1 [pg/ml] day 17 | = | **0.001** | **0.019** | **0.002** | **<0.001** |
| TNFα [pg/ml] day 3 | 5.5 (1.5;7.1) | 0.714 | 0.844 | 0.960 | 0.272 |
| TNFα [pg/ml] day 10 | = | 0.963 | 0.743 | 0.651 | 0.405 |
| TNFα [pg/ml] day 17 | = | 0.306 | 0.108 | 0.912 | 0.308 |
